# *Sox9* Expression in Amniotes: Species-Specific Differences in the Formation of Digits

**DOI:** 10.3389/fcell.2017.00023

**Published:** 2017-03-23

**Authors:** Juan A. Montero, Carlos I. Lorda-Diez, Javier Francisco-Morcillo, Jesus Chimal-Monroy, Juan A. Garcia-Porrero, Juan M. Hurle

**Affiliations:** ^1^Departamento de Anatomía y Biología Celular and IDIVAL, Universidad de CantabriaSantander, Spain; ^2^Departamento de Anatomía, Biología Celular, y Zoología, Universidad de ExtremaduraBadajoz, Spain; ^3^Departamento de Medicina Genómica y Toxicología Ambiental, Instituto de Investigaciones Biomédicas, Universidad Nacional Autónoma de MéxicoMexico City, Mexico

**Keywords:** limb development, interdigit regression, chondrogenesis, skeletal progenitors, SOX9 transcription factor

## Abstract

In tetrapods the digit pattern has evolved to adapt to distinct locomotive strategies. The number of digits varies between species or even between hindlimb and forelimb within the same species. These facts illustrate the plasticity of embryonic limb autopods. *Sox9* is a precocious marker of skeletal differentiation of limb mesenchymal cells. Its pattern of expression in the developing limb has been widely studied and reflects the activity of signaling cascades responsible for skeletogenesis. In this assay we stress previously overlooked differences in the pattern of expression of Sox9 in limbs of avian, mouse and turtle embryos which may reflect signaling differences associated with distinct limb skeletal morphologies observed in these species. Furthermore, we show that *Sox9* gene expression is higher and maintained in the interdigital region in species with webbed digits in comparison with free digit animals.

The limb is an excellent model system to study the molecular basis of morphogenesis (Hinchliffe, 2002; Fabrezi et al., 2007). The skeletal pattern of the limb is conserved in tetrapods, yet differences in bone morphology are remarkable among different species (Kavanagh et al., 2013). Interpretations of skeletal limb diversification has been largely based on comparative developmental studies using histochemical or radiolabeling markers of initial stages of cartilage differentiation. From these approaches it has been proposed that the limb skeleton in tetrapods is generated by sequential branching and segmentation of a basic pattern representative of the distal segment of the fish fins, termed the “metapterygial axis.” The advent and progress of molecular biology has provided new insights about the diversification of the limb skeletal morphology. For example, it has been shown that activation of signals responsible for skeletogenesis may be differentially regulated by transcriptional enhancer DNA sequences that are species-specific (Kvon et al., 2016). These studies explain major skeletal differences in evolutionary distant species such as the absence of limbs in snakes. However, differences between the fore- and the hind-limb in the same species or skeletal differences observed among closely related tetrapods might be regulated in a different fashion, such as timing differences in the expression of signaling molecules (Richardson et al., 2009; Moore et al., 2015; Zuniga, 2015).

Sox9 is a well known marker of the skeleton that precedes the appearance of cartilage blastemas (Wright et al., 1995; Healy et al., 1999; Chimal-Monroy et al., 2003; Kawakami et al., 2005; Lorda-Diez et al., 2011; Sensiate et al., 2014). Hence, Sox9 is expressed even in domains that represent skeletal pieces lost in the course of evolution of specialized species (de Bakker et al., 2013). Silencing Sox9 in mouse embryos causes loss of appendicular skeleton and increases programmed cell death (Akiyama et al., 2002). Sox9 overexpression promotes polydactyly (Akiyama et al., 2007). Furthermore, Sox9 along with BMP and WNT signaling are considered key regulators of digits formation through a self-organizing Turing mechanism (Raspopovic et al., 2014). Overall, such findings make Sox9 an excellent marker to detect signaling differences, later transduced into specific patterns of chondrification (Richardson et al., 2009), responsible for variations in the morphology of the appendicular skeleton. Based on the observation of *in situ* hybridizations, we have revised the pattern of Sox9 gene expression during digit development in reptilian (Mauremys turtle), avian (chick and duck), and mammal (mouse) species with different autopodial morphology to uncover signaling differences of potential interest to explain digit morphogenesis.

In chick embryos the expression of *Sox9* shows differences between the wing (Figures 1A–G) and the leg bud (Figures 1H–M). In wing buds at stage HH22 (3.5 id) the expression of *Sox9* marks the primary axis of the appendicular skeleton. In next stages, the initial domain extends proximally and distally (Figures 1A–C). Proximally, the domain forms the humerus primordium, and distally it shows a branching that establishes the primordium of the radius (Figures 1C,D). By stage HH24 (4 id) the primary axis is continued distally by the digital arch oriented toward the anterior margin of the bud. Between stages HH26-HH28 the digital arch undergoes a branching process to form each digit (Figures 1E–G). First branching forms digit 3 and a common branch that bifurcates to form digit 4, and a reduced domain reminiscent of a digit 5. The latest, is progressively reduced in size and expression intensity. The most anterior digit, is formed distally and aligned with the radial domain (Figures 1F,G).

**Figure 1:**
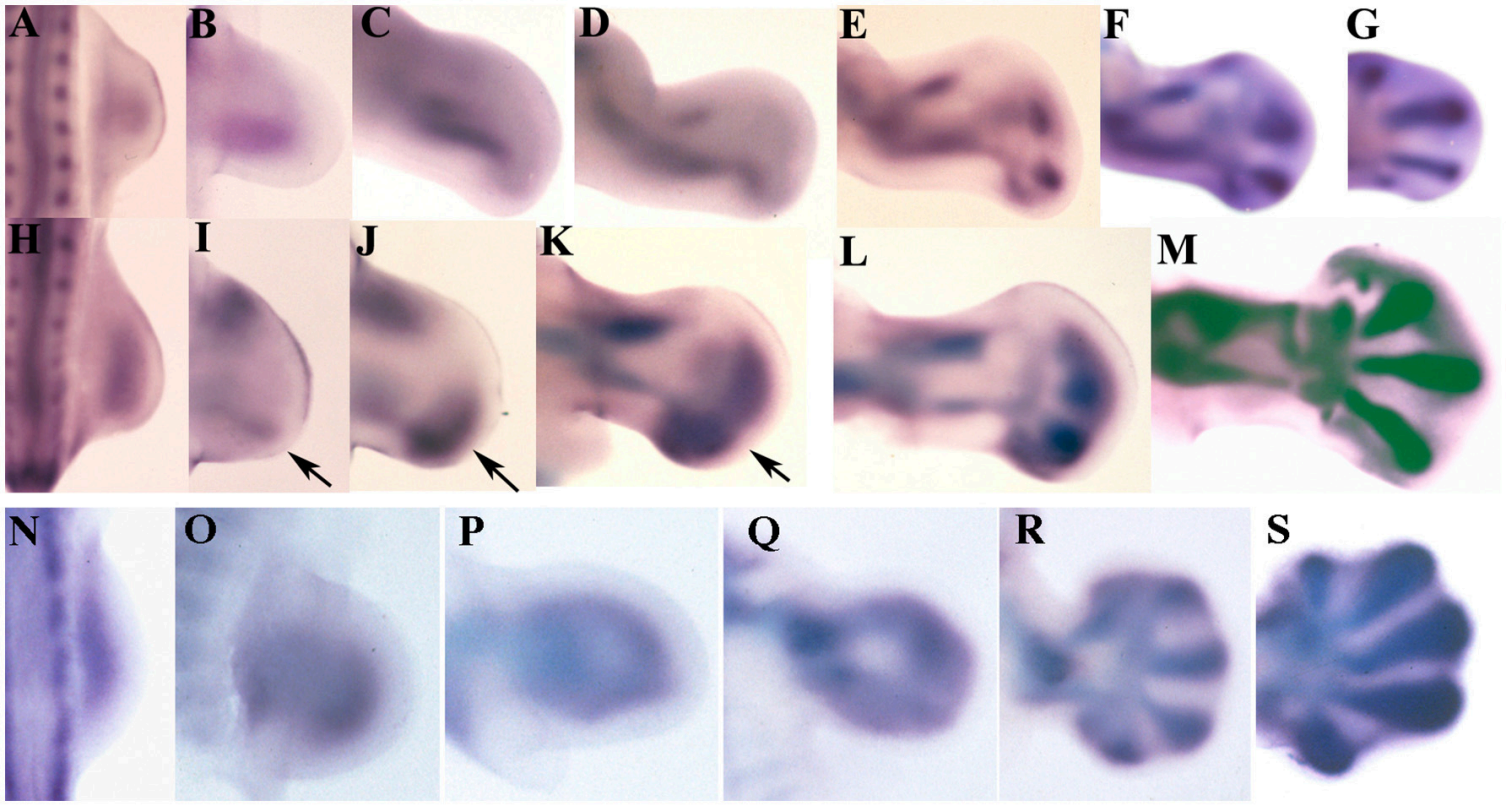
*****Sox9*** expression during limb development of chicken and mouse embryos. (A–G)** Embryonic chicken wing buds at stages HH20 **(A)**, HH22 **(B)**, HH24 **(C)**, HH 25 **(D)**, HH26 **(E)**, HH27 **(F)**, and HH28 **(G)**. **(H–M)** Chicken leg buds at stages HH20 **(H)**, HH22 **(I)**, HH23 **(J)**, HH25 **(K)**, HH27 **(L)**, HH28 **(M)**. Arrows indicate the position of the digital arch domain. **(N–S)** Mouse forelimbs illustrating the sequence of *Sox9* expression at stages E9,5 **(N)**, E10 **(O)**, E10,5 **(P)**, E11 **(Q)**, E12 **(R)**, and E13 **(S)**. Anterior is to the top and distal to the right in all images.

In the leg bud the initial expression of *Sox9* at stage HH22 appears divided into a posterior (primary axis) and an anterior domain for the tibia (Figures 1H,I). The femur is identifiable at stage HH25 coupled between the proximal end of the fibular and tibial domains (Figure 1K). The appearance of these skeletal domains at stage HH23 is accompanied by the formation of a nascent digital arch that occupies a posterior and distal position (Figures 1K,L). Initially, the expression is uniform and limited to the posterior half of the autopod but, in the following stages (HH25 and HH26), the expression progresses anteriorly and digits became identifiable as patches of higher expression (Figures 1J–M). Digits 3 and 4 are the most prominent at these stages while digits 2 and 5 are poorly defined areas where the expression of Sox9 is not very intense. Interestingly, the most anterior part of the autopod lacks *Sox9* transcripts until stage HH26-HH27.

Both in the wing and in the leg bud, concomitantly with the intensification of *Sox9* expression at stage HH26 in the digit blastemas, a carpal/tarsal arch of lower *Sox9* expression level is formed. Carpal and tarsal pre-cartilages are individualized when digit blastemas are defined.

Expression of *Sox9* in the mouse is similar in fore- and hind-limbs (Figures 1N–S). Initial expression of *Sox9* occupies the whole central region of the early bud (Figures 1N,O). Regionalization of this domain in stylopod, zeugopod and digital arch is due to the loss of transcripts from the central region at 10.5 pc (Figures 1P,Q). Due to this process, expression of *Sox9* appears as a loop where the distal curved region constitutes the digital arch. The proximal part of the loop lengthens marking the position of the stylopod. The zeugopodial elements are identified as the lateral regions of the loop. In next stages the digit primordia appear as elongated domains of intensified *Sox9* gene expression (Figures 1R,S). Digits 3 and 4 are the first to appear.

The skeletal domains of *Sox9* in the Mauremys turtle are similar in fore and hind-limbs (Figure 2). At the beginning, a central ill-defined domain is transformed into a triangular domain with a posterior elongated vertex, which marks the stylopod (Figure 2C). The sides of the triangle form the zeugopodial domains, and the base corresponds with the digital arch. The expression of Sox9 in the digital arch becomes progressively intensified at discrete regions to form digit primordia (Figures 2D,E). Digits 3 and 4 are the most precociously identifiable while digit 1 is the last to appear, preceded by digit 5 (Figures 2E–I). In the course of digit development, the expression of Sox9 is progressively restricted to the digit tip and to the developing joints (Figures 2E–I).

**Figure 2:**
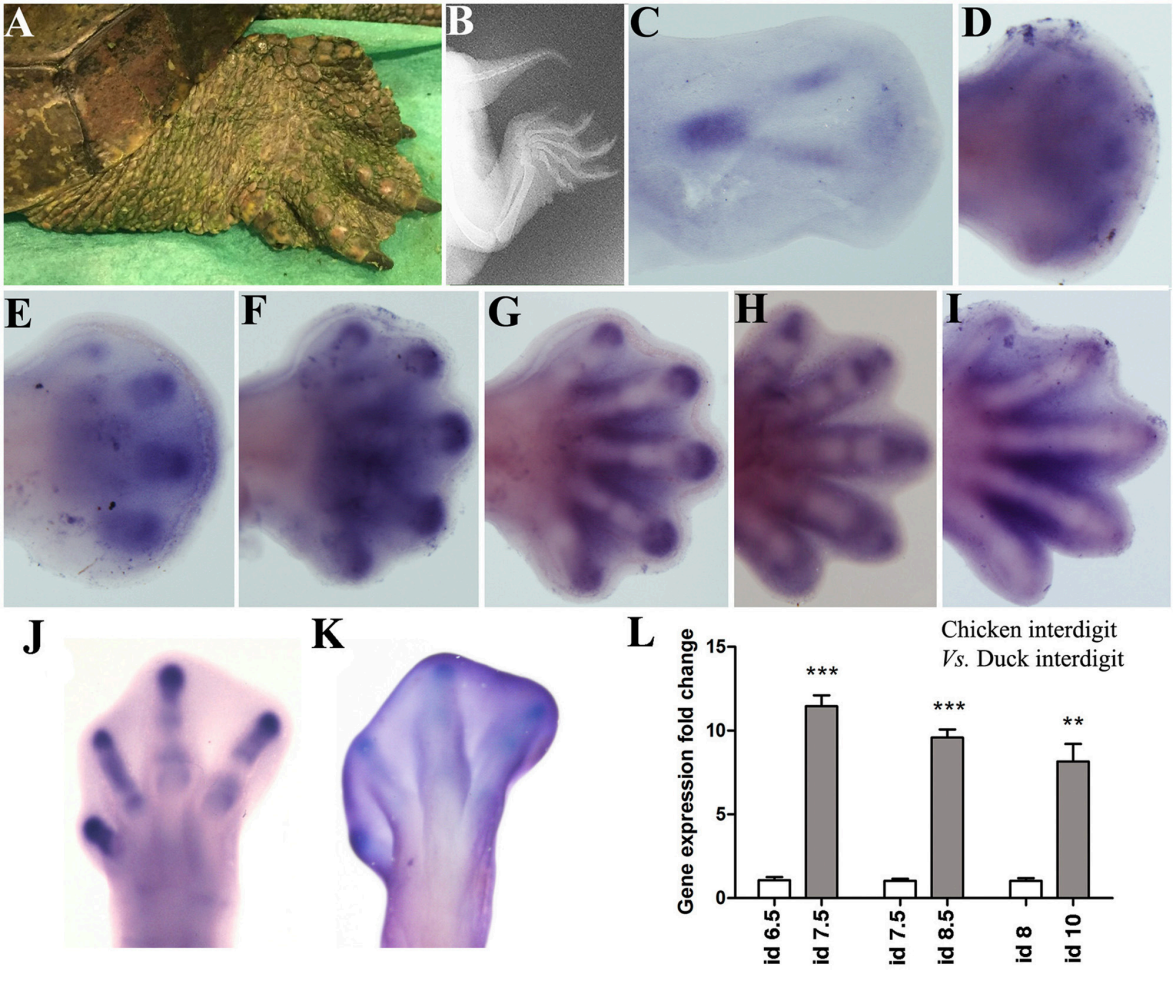
*****Sox9*** expression during limb development in the turtle (***Mauremys Leprosa***)**. Stages were established according to the developmental series of Yntema (1968). **(A,B)** adult limb in a live picture **(A)** and a radiographic image **(B)** to illustrate the presence of interdigital membranes in this species. **(C–I)** *Sox9* expression at stages Y14 **(C)**, Y15 **(D)**, Y16 **(E)**, Y17 **(F)**, Y18 **(G)**, Y19 **(H)**, and Y20 **(I)**. **(J–L)** Comparative analysis of *Sox9* expression in chick and duck interdigits. **(J,K)** *Sox9* expression in the chicken and duck autopod at day 6.5 and 8 of incubation respectively. **(L)** QPCR comparison of *Sox9* expression level in the developing third interdigit of the leg bud of chicken (white bars) and duck (gray bars) embryos at equivalent developmental stages. Each value represents the mean of three samples of 12 interdigits and statistical significance was set at *P* < 0.05. Incubation days (id) from left to right: chicken id 6.5 vs. duck id 7.5; chicken id 7.5 vs. duck 8.5; and chicken id 8 vs. duck id 10. Q-PCR specific primers were designed searching for identical homologous sequences in the duck and chicken for Sox9 and GAPDH genes. ^**^p ≤ 0.01; ^***^p ≤ 0.001.

Remarkably, the Mauremys turtle interdigital regions retain considerable levels of *Sox9* expression not observed in chick and mouse embryos (Figures 2F–I). To ascertain if interdigital expression of *Sox9* associates with the absence of interdigit remodeling in the Mauremys turtle (Figure 2A), we compared the level of expression of *Sox9* in the third interdigit of the leg bud of chick and duck embryos, as characteristic models of species with free and webbed digits respectively. As shown in Figures 2J–L, expression of *Sox9* in the non-regressing interdigit of the duck was much higher than that of the chick embryo.

Detailed analysis of phylogenetically related but phenotypically different species have provided important cues about the mechanisms accounting for limb morphogenesis (Moore et al., 2015). Gene expression computational modeling have also provided insights on the molecular bases responsible for differences in limb skeletogenesis among vertebrates (Uzkudun et al., 2015). The consideration of the subtle *Sox9* expression differences highlighted in this “perspective” assay, are consistent with heterochrony detected in the stages of chondrification (Richardson et al., 2009), and may help to improve our understanding of how digits differ in morphology. In all species, the expression of *Sox9* marks the successive appearance of the stylopod, zeugopod, and autopod along the proximo-distal axis of the limb. The autopod includes the mesopodium (carpi/tarsi) and the acropod (digits); the specification of zeugopod and the acropod has been proposed to determine the mesopodial intermediate domain in between (Woltering and Duboule, 2010). Consistent with this hypothesis, the appearance of intensified expression of Sox9 marking the nascent digits precedes that of the carpal/tarsal domains that lie in the concavity of the digit arch.

The formation and expansion of the digit arch in the chick embryo is clearly distinct from that observed in mouse and turtle embryos. Consistent with the evolutionary model proposed by Shubin and Alberch (1986), in the avian limb the progressive appearance of the digit expression domains follows a polarized sequence from posterior to anterior, which is more accentuated in the wing bud. In contrast, the digital arch domain in mouse and turtle limbs appears occupying a central position in the autopod, and in the course of development expands uniformly to the margins of the bud. These differences raise doubts about the validity of current thought, which considers independent identities for each of the digits in the hand/foot of vertebrates. The consideration of such identities have implications for evolutionary hypothesis that consider digit 1, as the most distal element of a conserved skeletal axis modified in the course of evolution through branching and segmentation processes (see Cohn et al., 2002, for discussion). Sox9 expression domains precede the appearance of prechondrogenic blastemas that were formerly employed in traditional comparative embryonic studies. Hence, the differences among species observed here, support mechanisms of skeletal diversification based on the combination of a distinct distribution of signals with differences in the intrinsic properties of the skeletal progenitors of the autopod, likely associated with differential epigenetic signatures (Sheth et al., 2016). Both in the wing and in the leg of avian embryos, digits are different along the antero-posterior axis justifying the consideration of different digit identities according to their position and number of phalanxes. In contrast, digits of mouse and turtle embryos, expands from the centrally located digital arch toward the margins of the limb bud. In this model of digit arch expansion, there are not morphological landmarks that allow to assign specific identities to the central digits (2,3,4). It must be taken into account that the carpal/tarsal domains of *Sox9* appear when the digit arch shows independent digit domains. Therefore, at these embryonic stages mesopodial domains cannot be taken as a primary reference to establish the identity of the digits. The only morphological differences observed among the pre-cartilaginous blastemas are located in the marginal digits (digits 1 and 5) where *Sox9* domains exhibit a reduced size and appear at more advanced stages than the central digits.

The growth of the limb bud is regulated by a complex signaling network (Uzkudun et al., 2015), where *Shh* and *Gremlin1* genes play an important role in digit specification (Sanz-Ezquerro and Tickle, 2003a; Zhu et al., 2008). Evolutionary or genetic deregulations of the *Shh/Gremlin* loop causes polydactylous (Norrie et al., 2014) or oligodactylous (Lopez-Rios et al., 2014) autopods. Consistent with our interpretation, central digits in these mutants, regardless of its number, are identical and indistinguishable from each other (Norrie et al., 2014). These findings make plausible that digit formation result of the self-organization of the limb mesenchyme (Cooper, 2015), within an autopod of dimensions and shape finely tuned by regulatory genes responsible for growth (Zhu et al., 2008).

Sox9 is target of signals controlling proliferation and differentiation of the skeletal progenitors, including FGFs, BMPs, TGFbetas, and Retinoic acid (RA). These signals are themselves closely regulated by the AER and the ZPA, to establish the pattern of limb skeletogenesis as well as the number of digits in the autopod. BMPs up-regulate the expression of *Sox9* and promote differentiation of progenitors (Lorda-Diez et al., 2014; Norrie et al., 2014) and in conjunction with TGFβs and Activins induce the formation of extra-digits in the avian limb (Chimal-Monroy et al., 2003; Montero et al., 2008). FGFs are major determinants of digit size (Sanz-Ezquerro and Tickle, 2003b; Seki et al., 2015). FGFs inhibit chondrogenesis but expand the amount of Sox 9 positive skeletal progenitors and its overexpression in the limb results in the formation of extra cartilages, including extra-digits (Montero et al., 2001; Norrie et al., 2014), or extra-phalanxes (Sanz-Ezquerro and Tickle, 2003b). RA is a potent inhibitor of Sox9 gene expression (Weston et al., 2002) and RA inhibition in the autopod causes the formation of extra digits (Rodriguez-Leon et al., 1999). Hence, the pattern of Sox9 gene expression may reflect differences in the spatial distribution of signals within the limb bud mesoderm. According with this interpretation, avian digits may represent an evolutionary specialization of digit development consequence of a posterior polarization of signals responsible for limb outgrowth. In contrast, the pentadactyl autopod of mouse and turtle embryos may result from the uniform expansion (like opening a fan) of the signals that coordinate proliferation and differentiation of the skeletal progenitors.

## Ethics statement

The animal care and handling, and all the experimental procedures were in accordance with the guidelines of the European Communities Council and the Spanish legislation and they were approved by the Service of Animal Health and Welfare of the Regional Government of Cantabria (Reference No. PI-10-15).

## Author contributions

Conceived and designed the experiments: CL, JF, JG, JC, and JH. Performed the experiments: CL, JM, JF, JC, and JH. Analyzed the data: CL, JM, JG, JC, and JH. Writing of the manuscript: JM and JH.

### Conflict of interest statement

The authors declare that the research was conducted in the absence of any commercial or financial relationships that could be construed as a potential conflict of interest.
